# Unbiased Data Analysis for the Parameterization of Fast Translocation Events through Nanopores

**DOI:** 10.1021/acsomega.2c00871

**Published:** 2022-07-19

**Authors:** Florian L. R. Lucas, Kherim Willems, Matthijs J. Tadema, Katarzyna M. Tych, Giovanni Maglia, Carsten Wloka

**Affiliations:** † Groningen Biomolecular Sciences and Biotechnology Institute, University of Groningen, 9712 CP Groningen, The Netherlands; ‡ IMEC, Kapeldreef 75, B-3001 Leuven, Belgium; § Lab for Nanobiology, Department of Chemistry, KU Leuven, 3001 Leuven, Belgium

## Abstract

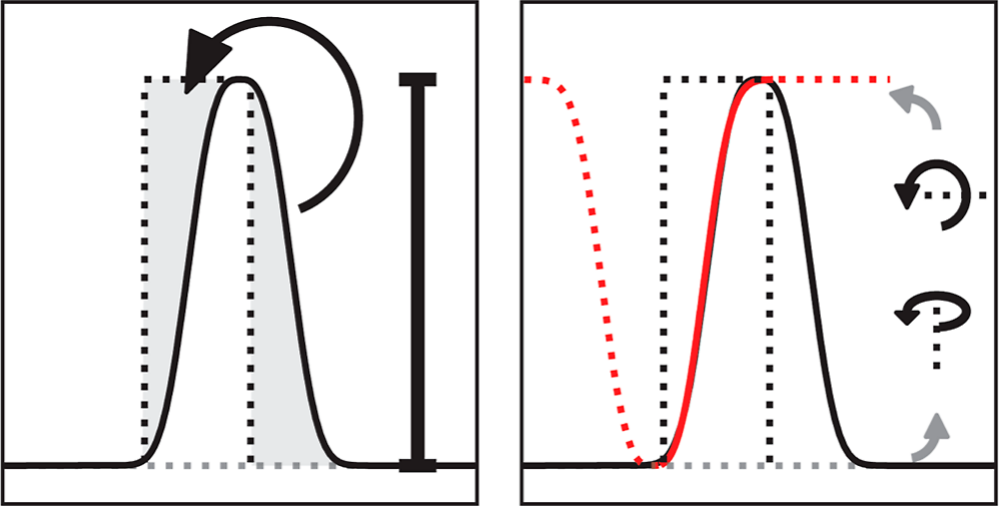

Single-molecule nanopore electrophysiology is an emerging technique for the detection of analytes in aqueous solutions with high sensitivity. These detectors have proven applicable for the enzyme-assisted sequencing of oligonucleotides. There has recently been an increased interest in the use of nanopores for the fingerprinting of peptides and proteins, referred to as single-molecule nanopore spectrometry. However, the analysis of the resulting electrophysiology traces remains complicated due to the fast unassisted translocation of such analytes, usually in the order of micro- to milliseconds, and the small ion current signal produced (in the picoampere range). Here, we present the application of a generalized normal distribution function (gNDF) for the characterization of short-lived ion current signals (blockades). We show that the gNDF can be used to determine if the observed blockades have adequate time to reach their maximum current plateau while also providing a description of each blockade based on the open pore current (*I*_O_), the difference caused by the pore blockade (Δ*I*_B_), the position in time (μ), the standard deviation (σ), and a shape parameter (β), leaving only the noise component. In addition, this method allows the estimation of an ideal range of low-pass filter frequencies that contains maximum information with minimal noise. In summary, we show a parameter-free and generalized method for the analysis of short-lived ion current blockades, which facilitates single-molecule nanopore spectrometry with minimal user bias.

## Introduction

**Figure 1:**
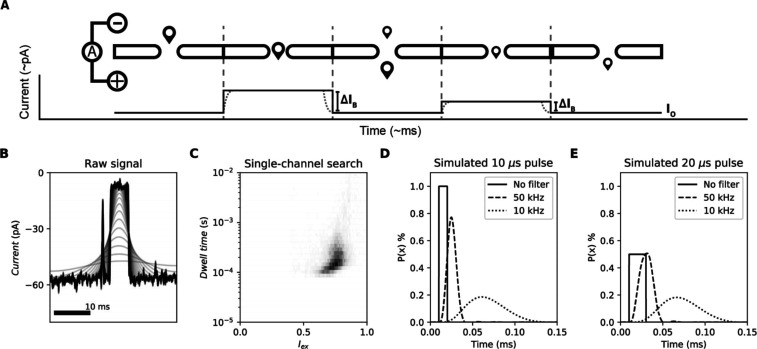
Nanopore experiments, dwell time skewness, and simulated pulse-dilated ideal pulses. (A) Schematic representation of nanopore electrophysiology. A measurement potential is applied across a nonconductive membrane with a nanometer-sized aperture inserted. A stable current can be measured across the open pore, and, upon entry of an analyte, the current is reduced relative to the ion exclusion caused by the analyte. (B) Raw current trace of 10 μM (added to *cis*) of a pentapeptide (YAGFL) measured in 1 M KCl, pH 4.2 buffered using 15 mM citric acid, and bis-tris-propane under an applied potential of negative 100 mV (*cis–trans*) at a sampling frequency of 500 kHz with a recording bandwidth of 100 kHz and filtered between 25 Hz and 5 kHz, each represented with a separate line using a digital Gaussian filter using fragaceatoxin C modified with a tryptophan at position 13 (G13W-FraC). (C) Excluded current set against the dwell time for a measurement performed as in part A. For (A,B), experiments were performed using an Axon 200B amplified (molecular devices) coupled to a Digidata 1550B (Molecular Devices). Event characteristics were determined using the single-channel search method as implemented in ClampFit 10. The events were exported to an Excel sheet and plotted using matplotlib (Python 3.9). (D) A 10 μs pulse is shown together with the resulting pulses after 50 and 10 kHz 4-pole Bessel filtering. (E) A 20 μs pulse is shown together with the resulting pulses after 50 and 10 kHz 4-pole Bessel filtering. Simulation was performed using a continuous-time linear system (lsim2) as implemented in SciPy on ideal pulses sampled at 100 MHz.

Single-molecule nanopore electrophysiology is an emerging field with the most notable application in label- and amplification-free nucleic acid sequencing, enabling the identification of long stretches of nucleic acids as they translocate through a nanopore.^1−4^ The working principle is similar to nanometer-sized Coulter counters in allowing the differentiation between nucleotides (and other molecules) due to differences in ion displacement (Figure 1A).^5^ The current generation of nanopores is now capable of detecting individual amino acids,^6^ opening a new frontier for the sequence identification of proteins. However, whereas nucleic acid sequencing typically needs to differentiate between four bases, proteins can consist of 20 or more amino acids with widely diverging sizes and charges. A helicase attached to the top of the nanopore can be used to aid the translocation of uniformly charged nucleic acids, whereas due to the variability of the protein samples, alternative strategies are required. Even though proof-of-concept studies have shown that motor proteins can be used to control translocation of peptides across nanopores,^7−10^ they are currently not able to resolve functional sequence information. Alternatively, proteins can be identified “bottom-up”, based on their fingerprint following a trypsin digest, as commonly done in mass spectrometry experiments.^11−13^

When a nanopore has a voltage applied across it, an ionic current can be measured, which directly relates to the physicochemical properties of that specific pore type, that is, its size and the charge distribution on its surface. A reduction in this measured ionic current, attributed to the entry of a molecule into the nanopore, is termed an “event” (Figure 1B). Fast translocation events have been historically dealt with by modifying nanopores chemically,^14^ and to build fast and accurate current amplifiers,^15−18^ events resulting from a tryptic digest of a protein should correspond to the entry and translocation of individual peptides through the pore. In order to identify events generated by the entry of molecules into a nanopore, the following analysis steps are generally required: (1) preprocessing, involving, for example, smoothing of the data, and removal of instrument noise, (2) event localization, that is, “when does an event occur”, which is commonly performed using a threshold search algorithm, and (3) event characterization, involving the extraction of event characteristics based on the fluctuations observed during an event. It is hypothesized that additional information from short-lived events contained within individual noise components, such as frequency fingerprinting or a Fourier transform, could allow individual characterization of molecules.^19−22^

The potential for obtaining additional information about the sample from short events occurring near the noise floor of the raw data creates a need for critical preprocessing and characterization. Optimal preprocessing is an intricate problem as the residence time of peptides inside the nanopore is generally in the order of micro- to milliseconds, and the change in signal induced by the current blockade is in the picoampere range. The root-mean-square noise of commonly used amplifiers is close to the same order of magnitude, creating a relatively low signal-to-noise ratio.^23,24^ Thus, a preprocessing filter is nearly always a necessity. However, if the effective filter frequency of the event reaches the same order of magnitude as the residence time of an event, we observe that the blocked current appears reduced, while the residence time appears elongated. While the determination of the event localization can also be considered a preprocessing step in many applications, it does not transform the data and therefore requires less accuracy.

Here, we present a universally applicable method for the characterization of short-lived events, where the required sampling frequency to correctly capture the amplitude of the current blockade for each event can be calculated and verified. Herein, events are characterized based on a generalized normal distribution function (gNDF), which allows the description of events based on the open pore current (*I*_O_), difference caused by the blocked pore (Δ*I*_B_), position in time (μ), standard deviation (σ), a shape parameter (β), and the noise component. Additionally, this method allows the estimation of an ideal range of low-pass filter frequencies that contain maximum information with minimal noise. Taken together, this may provide a direct correlation between different data sets, independent of the measurement setup used.

## Results and Discussion

### Assumptions for Nanopore Event Detection

Generally, events are described as the relative blocked current [i.e., the residual current (*I*_res_) or its complement to one: the excluded current (*I*_ex_)] and residence time (or dwell time). The description of events based on the residual current, as a direct result of analytes translocating pores, is subject to several assumptions:

#### Assumption 1

The diameters of the pore and analyte, conductivity of the buffer solution, and length of the pore remain constant (Supporting Information 1).^25^

#### Assumption 2

Because the nanopore acts as a resistor–capacitor circuit (RC circuit), we assume that the residence time of peptides inside the nanopore must be larger than the required time to charge the RC circuit (expressed as the RC time constant, or τ_system_). We typically observe a time constant in the microsecond range. This can be corrected under assumption 1 and expressed as eq 1
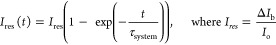
1where *I*_res_ (*t*) is the residual current at time point *t* and *I*_res_ is the residual current, equal to the fraction of the difference in current caused by the block Δ*I*_b_ and the open pore current *I*_o_. It is important to note that one can only measure the residual current at a given time point *t.* If this assumption is not satisfied, an increase in the residual current should be observed. However, the dwell time of an event should remain (roughly) constant.

#### Assumption 3

The current blockade does not have any bandwidth limitation. Current blockades are, however, always filtered as we observe a physical translocation through the nanopore, which is not instant. Thus, we describe short events as ideal pulses, which, in the process of the measurement, are dilated by filtering effects. We utilize the term filtering effects here to describe all phenomena causing divergence from an ideal pulse. Additionally, it is important to note that the effective sampling frequency is not always equal to the sampling frequency set during acquisition as events may be dilated by physical events, such as aliasing, analog-to-digital converter limitations, and the capacitance induced by the system. When the event duration reaches or is briefer than the effective sampling period, the event is broadened, and the dwell time, and therefore the excluded current, becomes probabilistic. Effectively, this means that the observed current is lower than the actual current block caused by the analyte, while the observed dwell time is increased.

We compare three representative algorithms to understand several artifacts that arise from these assumptions. The first algorithm is the Fetchan algorithm as implemented in pCLAMP (Molecular Devices), which considers assumptions 1, 2, and 3 to hold. For long-lived events, we notice that all assumptions hold. However, when the residence time of peptides is short, assumption 3 is not fulfilled. This is observed as an excluded current “tail”, where the excluded current seems to decrease while the dwell time is increased, which is biased toward the minimal observable residence time and therefore seems centered (Figure 1C). We visualize this effect using simulated data by application of a commonly used 4-pole (digital) Bessel filter.^26^ When a 10 μs pulse (100 kHz) is filtered to 50 kHz, we notice that the event area remains equal, while the dwell time increases and the excluded current decreases (Figure 1D). When a 20 μs pulse (50 kHz) with half the excluded current undergoes the same 50 kHz filter, the excluded current is not decreased (Figure 1E).

The adaptive time-series analysis (ADEPT) by Balijepalli et al. considers assumption 1 and corrects for the capacitance in the system.^25^ In ADEPT, the excluded current is represented as a Heaviside step function multiplied by the rise and fall time based on the RC time constant and considers that multiple pulses may be present in each blockade. Notably, the Heaviside step implicates that assumption 3 holds; however, the authors consider that the Heaviside step function could be replaced in future.

Lastly, we investigated the second-order-differential-based calibration (DBC) method,^27^ as proposed by the Long group, which is able to accommodate any shape of an event^27^ and, by extension, seems independent of assumptions 1, 2, and 3. The method attempts to correct the dwell time of a blockade based on the area under the event, which is (for the most part) not dilated. The DBC method assumes that the event has reached the full height. However, it is difficult to validate this assumption, as we exemplified for 10 μs and 20 μs pulses, which appear equal when they are filtered to 10 kHz (Figure 1D,E); therefore, the accurate determination of the dwell time and excluded current becomes virtually impossible.

### Event Characterization

We hypothesized that a method combining ADEPT with DBC would allow event characterization relying merely on assumption 1. We sought to implement a well-defined function that assumes the shape of any event. To allow the full description of events with five parameters leaving only the noise component, we propose the use of a gNDF, *f*(*x*; *I*_O_,Δ*I*_B_,σ,β,μ), for the optimization of events (eq 2)
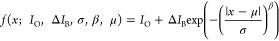
2with *x*, the dependent variable (e.g., the time), *I*_O_, the open pore current, Δ*I*_B_, the difference in current of the event relative to *I*_O_, β, the shape parameter, σ, the standard deviation, and μ, the location. In essence, the probability function is multiplied by the blocked pore current (Δ*I*_B_) to signify the observed event. The probability density function (PDF) of the gNDF is well characterized and shown in eq 3
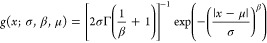
3

The CDF is given by eq 4:
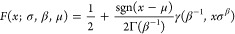
4with Γ, the gamma function, and γ, the unnormalized incomplete lower gamma function. The quantile function (inverse of the CDF) is given by eq 5
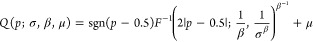
5with *p*, the probability. Finally, the full width at half-maximum (fwhm) is given by eq 6
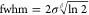
6

The resulting function assumes the shape of an ideal pulse, equal to the ideal pulse of a Heaviside step function when the exponent β reaches infinity while displaying a Gaussian profile when this parameter equals 2. In previous contributions, we utilized a similar function for the detection and exclusion of events based on their shape;^12,13^ however, it is important to note that the gNDF in this contribution follows a slightly modified version.

**Figure 2:**
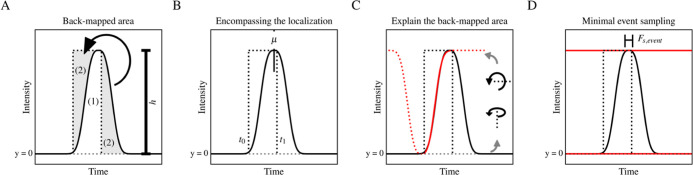
Conceptual representation of events described in this article. Each figure shows a gNDF as a continuous solid black line with β = 3, σ = 1, and μ = 0. The black dashed lines represent the reconstructed ideal pulse event. The gray dashed line represents the baseline at *y* = 0. (A) Back-mapped area where the gray patches (2) represent the same area. The arrow indicates that the area after the event has the equal area as the missing area of the event’s ideal pulse. (1) indicates the area under the observed event. (B) shows that the dwell time of an event must encompass the localization of the fitted function. The solid vertical line indicates the event localization (μ). (C) shows that the back-mapped curve must overlap with the forward event. The red dotted line represents the inverse of the gNDF shifted by the dwell time, and the solid red part of this line represents the overlap. (D) shows the manual event frequency, where the top red lines represent the probability thresholds (*p* = 0.001) at the top and bottom of the event. The indicator line with the two solid vertical lines shows the range of the curve where the difference in the cumulative distribution function (CDF) with respect to the localization is equal to the probability threshold.

The length of events resulting from protein or peptide translocation is one of the most important features that can be extracted from a signal. Of major concern, for short-lived events, is the skewness in the estimated dwell time due to filtering effects. An interesting approach taken by Long and co-workers utilizes the area under the signal as this is almost fully independent of the effective sampling period.^27^ However, the total area encompassed by an ideal event is assumed to be equal to the event length multiplied by the event height. As the dwell time approaches the effective sampling period, pulse dilation is expected such that the observed event height is reduced and the observed event length is increased.^26,28^ In order to derive the characteristics of an event, we first consider that it reaches the true amplitude of the pulse. In this scenario, we can determine the dwell time of an event by reconstructing the ideal pulse. We know that the area under the event from the ideal pulse should be equal to the area under the fitted event, as expressed in eq 7 and visualized in Figure 2A.
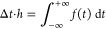
7where Δ*t* is the total event time, *h* is the event height, and *f*(*t*) is the probability density of the gNDF at any given time *t*.

By utilizing the maximum ordinate of the gNDF, we can derive the total event time (Supporting Information 2), resulting in eq 8
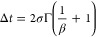
8where Δ*t* is the total event time, β is the shape parameter, σ is the standard deviation, and Γ denotes the incomplete gamma distribution.

Importantly, we postulate that eq 8 is only quantitative if the observed event encompassed the localization (μ, Figure 2B), which can be easily validated (Supporting Information 3). Moreover, we postulate that eq 8 is also only valid if the event area can be explained completely by the back-mapped area. We can validate this by comparing the probability at half-width at half-maximum with the complement to one equidistant probability after *t*_1_ (Supporting Information 4, Figure 2C). Interestingly, this provides a singular, nonanalytically solvable, probability threshold for the detection of *t*_0_. However, the true event height must be validated differently
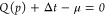
9where Δ*t* is the total event time, μ is the localization, and *Q*(*p*) denotes the quantile function at probability *p* (eq 5).

If there exists a value for *p* that satisfies eq 9, we can determine the quantitative limit of detection. As a result, we can determine the time an event is significantly occupying its current plateau (maximum ordinate). Thus, we calculate the effective frequency as the minimal observed change using eq 10 (Supporting Information 5, Figure 2D).
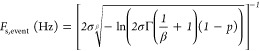
10where *F*_s,event_ is the effective frequency as the minimal observed change. It is important to note that the *F*_s,event_ is not necessarily related to the dwell time. Rather, it is a function of the shape of the observed event and is not defined when β reaches infinity. The gain (or loss) of the event amplitude can be calculated from the required sampling frequency of events (i.e., *F*_s,event_);^29^ however, as a rule of thumb, it is easiest to state that the sampling frequency must be equal or larger than the required sampling frequency of the event. Importantly, the second-order DBC method,^27^ as proposed by the Long group, is able to assume any shape of an event.^26^ However, it is difficult to validate the assumption that the maximum current blockade of an event is reached. Fortunately, *F*_s,event_ can be used to validate the DBC method and a possible conjunctive pipeline could be constructed based on a combination of the two to correct for (slight) variations in the shape of dilated events.

Additionally, usage of the fwhm (eq 6) is also justified for shorter events as the maximum difference between the integrated back-mapped dwell time is less than 1% when β reaches 2. These deviations are typically within the error margin of the fit to the data and therefore become virtually indistinguishable. We show the difference between the fwhm and back-mapped dwell time in Figure S1. Ultimately, this agrees with previous contributions, where the event dwell time is defined as starting on the vertical rising edge and ending on the descending edge of the signal pulse. The method presented in this contribution states that this assumption is correct if the shape parameter is large. It also explains the tail-like widening dwell time distribution seen for short pulses analyzed using “standard” single-channel search as the uncertainty in the estimated dwell time is increased. This effect is especially notable when used in, for example, pCLAMP’s Fetchan (Figure 1B) or the “rapid event detection” (RED) method, which we introduced in an earlier contribution.^30^

### Functional Implementation

We implemented the gNDF for fitting using a custom Python library (see code availability) suitable for the analysis of Axon Binary Files; however, other data loaders can be easily added. We implemented RED as described in the previous contribution^12,13,30^ and utilized it for event localization. Subsequently, we fit the gNDF around each observed event, after which the event features are determined as described in this contribution. All events are stored in a SQL file containing the start and end times of each event, the extracted features, and utilized fitting functions. The optimized results are stored in a separate table from RED, allowing validation of the results.

### Effect of Over- and Under-Filtering

**Figure 3:**
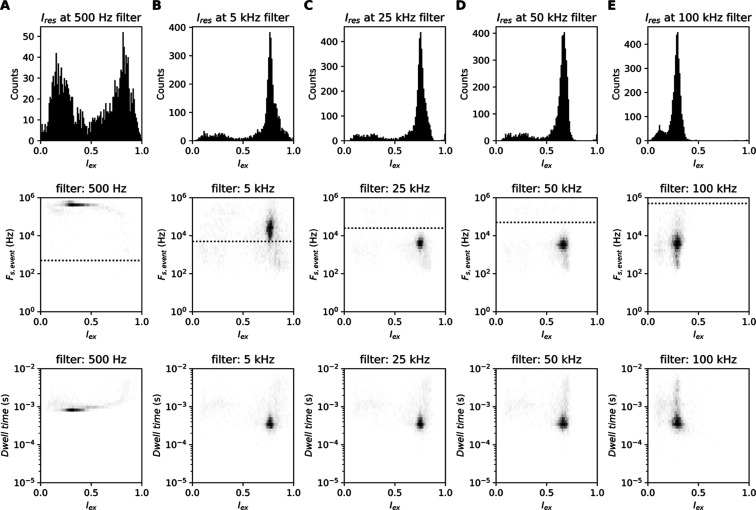
Optimized event detection using different cutoff filters. All panels represent the same data where a 10 μM (added to *cis*) of penta-peptide (YAGFL) was measured in 1 M KCl, pH 4.2 buffered using 15 mM citric acid, and bis-tris-propane under an applied potential of negative 100 mV (*cis*–*trans*) at a sampling frequency of 500 kHz with a recording bandwidth of 100 kHz using fragaceatoxin C modified with a tryptophan at position 13 (G13W-FraC). Experiments were performed using an Axon Axopatch 200B amplifier (Molecular Devices) coupled to an Axon Digidata 1550B (Molecular Devices). All events were localized using a 5 kHz Gaussian filter and subsequently characterized using a Gaussian filter at the filter frequency as described above each graph. (A–E) Top graph: the excluded current (*I*_ex_) of each observed event (*F*_s,event_ ≤ 500 kHz) binned in 200 residual current bins evenly distributed between 0 and 1 *I*_ex_. Middle graph: minimum required sampling frequency of events (*F*_s,event_) set against the *I*_ex_ for different Gaussian filter frequencies (see the title in each panel). The dotted line represents the filter frequency. Bottom graph: *I*_ex_ set against the integrated dwell time as estimated by eq 8 of this article for different Gaussian filter frequencies (see the title in each panel).

The over-filtering of events causes the observed excluded current to be lower than the expected value, while the observed dwell time is increased (Figures 1 and 3A). To minimize this artifact, we can localize events using a low frequency cutoff and characterize events with minimal filters. It may seem as if we can directly utilize unfiltered data; however, under-filtering may cause the observed excluded current to also be lower than the expected value as the baseline becomes indistinguishable from an event due to noise.^31^ We observe this effect when comparing the residual current histograms at different filter frequencies (Figure 3). Therefore, we postulate that there is a low-pass filter that can gain maximum information while minimizing noise. We pose that eq 8 holds under the assumptions that the origin of events can be described by immediate (ideal) pulses, all filter effects cumulatively result in a Gaussian-like shape, eq 9 is satisfied, and the event sampling frequency (eq 10) is equal or larger than the effective sampling frequency. We observe that *F*_s,event_ as calculated by eq 10 appears larger for signals that have been filtered at or under *F*_s,event_ and remains stable with higher frequency filters. If the filter frequency is lower than *F*_s,event_, we observe that the estimated dwell time is elongated (Figure 3A). When the effective filter frequency is (nearly) equal to or larger than *F*_s,event_, we observe the true dwell time (Figure 3B–E). This explains why a 5 kHz filter on the presented data results in a correct excluded current spectrum and dwell time, while the *F*_s,event_ appears larger. It is worth noting that for under-filtered events, while the excluded current changes due to baseline noise, the dwell time stays as expected.

## Conclusions

In this work, we presented a generally applicable function that can accurately describe data acquired by nanopore sensors with only five parameters with noise. We also show a novel method for the determination of the minimal required sampling frequency, which allows a reproducible data analysis pipeline, with minimal user bias.

For this, we use a gNDF. While the gNDF is a continuous function, we demonstrated that the events are finite. We showed that the gNDF can be used to determine the minimum required sampling frequency for an event to reach its maximum current plateau. Therefore, we have shown that the dwell time of events can be accurately determined using this method under the assumptions that the diameter of the pore and analyte, conductivity of the buffer solution, and length of the pore remain constant. While other approaches may be beneficial depending on experimental conditions, we presented a unified approach for the determination of nanopore event characteristics.

## Materials and Methods

### Data Recording

Recordings of ionic currents were obtained using an Axopatch 200B (Axon Instruments) combined with a Digidata 1550B A/D converter (Axon instruments), similar to the preceding work.^12,13^ The sampling frequency was set at 500 kHz for analyte recordings, the analog Bessel filter was set at 100 kHz, respectively. Data was recorded using Clampex 10 (Molecular Devices).

### Data Analysis

Data was analyzed using Jupyter Notebook (version 5.5.0) running with Python 3.6.5 (64-bit), both within the Anaconda (version 5.2.0) environment. Events were localized using a RED algorithm.^12,13,30^

### Data Availability

The authors declare that the data and code supporting the findings of this study are available within the article and its Supporting Information or from the corresponding authors upon reasonable request.

### Code Availability

The code is available under DOI: https://doi.org/10.5281/zenodo.6546320.

## Supporting Information Available

Extended mathematical information containing equations deriving the residual current at any given time, equations deriving dwell time from the gNDF, equations deriving event localization from the gNDF, equations deriving the one equidistant probability from the gNDF, equations deriving the minimal required sampling frequency from the gNDF, nanopore traces at different filter frequencies, difference in dwell time estimation when using the fwhm and back-mapped estimation, and histograms of fitted parameters for different filter frequencies (PDF)

## References

[ref1] Ayub M.; Bayley H. Individual RNA base recognition in immobilized oligonucleotides using a protein nanopore. Nano Lett. 2012, 12, 5637–5643. 10.1021/nl3027873. 23043363 PMC3505278

[ref2] Bayley H. Nanopore sequencing: from imagination to reality. Clin. Chem. 2015, 61, 25–31. 10.1373/clinchem.2014.223016. 25477535 PMC4404466

[ref3] Cao C.; Liao D.-F.; Yu J.; Tian H.; Long Y.-T. Construction of an aerolysin nanopore in a lipid bilayer for single-oligonucleotide analysis. Nat. Protoc. 2017, 12, 1901–1911. 10.1038/nprot.2017.077. 28837133

[ref4] Franceschini L.; Brouns T.; Willems K.; Carlon E.; Maglia G. DNA Translocation through Nanopores at Physiological Ionic Strengths Requires Precise Nanoscale Engineering. ACS Nano 2016, 10, 8394–8402. 10.1021/acsnano.6b03159. 27513592 PMC5221729

[ref5] Wanunu M. Nanopores: A journey towards DNA sequencing. Phys. Life Rev. 2012, 9, 125–158. 10.1016/j.plrev.2012.05.010. 22658507 PMC3780799

[ref6] Ouldali H.; Sarthak K.; Ensslen T.; Piguet F.; Manivet P.; Pelta J.; Behrends J. C.; Aksimentiev A.; Oukhaled A. Electrical recognition of the twenty proteinogenic amino acids using an aerolysin nanopore. Nat. Biotechnol. 2020, 38, 176–181. 10.1038/s41587-019-0345-2. 31844293 PMC7008938

[ref7] Manrao E. A.; Derrington I. M.; Laszlo A. H.; Langford K. W.; Hopper M. K.; Gillgren N.; Pavlenok M.; Niederweis M.; Gundlach J. H. Reading DNA at single-nucleotide resolution with a mutant MspA nanopore and phi29 DNA polymerase. Nat. Biotechnol. 2012, 30, 349–353. 10.1038/nbt.2171. 22446694 PMC3757088

[ref8] Brinkerhoff H.; Kang A. S. W.; Liu J.; Aksimentiev A.; Dekker C. Multiple rereads of single proteins at single-amino acid resolution using nanopores. Science 2021, 374, 1509–1513. 10.1126/science.abl4381. 34735217 PMC8811723

[ref9] Yan S.; Zhang J.; Wang Y.; Guo W.; Zhang S.; Liu Y.; Cao J.; Wang Y.; Wang L.; Ma F.; Zhang P.; Chen H.-Y.; Huang S. Single Molecule Ratcheting Motion of Peptides in a Mycobacterium smegmatis Porin A (MspA) Nanopore. Nano Lett. 2021, 21, 6703–6710. 10.1021/acs.nanolett.1c02371. 34319744

[ref10] Zhang S.; Huang G.; Versloot R. C. A.; Bruininks B. M. H.; de Souza P. C. T.; Marrink S.-J.; Maglia G. Bottom-up fabrication of a proteasome-nanopore that unravels and processes single proteins. Nat. Chem. 2021, 13, 1192–1199. 10.1038/s41557-021-00824-w. 34795436 PMC7612055

[ref11] de Lannoy C.; Lucas F. L. R.; Maglia G.; de Ridder D. In silico assessment of a novel single-molecule protein fingerprinting method employing fragmentation and nanopore detection. iScience 2021, 24, 103202 10.1016/j.isci.2021.103202. 34703997 PMC8521182

[ref12] Lucas F. L. R.; Sarthak K.; Lenting E. M.; Coltan D.; van der Heide N. J.; Versloot R. C. A.; Aksimentiev A.; Maglia G. The Manipulation of the Internal Hydrophobicity of FraC Nanopores Augments Peptide Capture and Recognition. ACS Nano 2021, 15, 9600–9613. 10.1021/acsnano.0c09958. 34060809 PMC8223486

[ref13] Lucas F. L. R.; Versloot R. C. A.; Yakovlieva L.; Walvoort M. T. C.; Maglia G. Protein identification by nanopore peptide profiling. Nat. Commun. 2021, 12, 5795 10.1038/s41467-021-26046-9. 34608150 PMC8490355

[ref14] Wanunu M.; Meller A. Chemically modified solid-state nanopores. Nano Lett. 2007, 7, 1580–1585. 10.1021/nl070462b. 17503868

[ref15] Rosenstein J. K.; Wanunu M.; Merchant C. A.; Drndic M.; Shepard K. L. Integrated nanopore sensing platform with sub-microsecond temporal resolution. Nat. Methods 2012, 9, 487–492. 10.1038/nmeth.1932. 22426489 PMC3648419

[ref16] Kim J.; Wang G.; Dunbar W. B.; Pedrotti K. An integrated patch-clamp amplifier for ultra-low current measurement on solid-state nanopore, 2010 International SoC Design Conference, Nov. 22–23 2010; IEEE, 2010; pp 424–427.

[ref17] Kim J.; Pedrotti K.; Dunbar W. B. An area-efficient low-noise CMOS DNA detection sensor for multichannel nanopore applications. Sens. Actuators, B 2013, 176, 1051–1055. 10.1016/j.snb.2012.08.075.

[ref18] Kim J.; Dunbar W. B. High-precision low-power DNA readout interface chip for multichannel nanopore applications. Sens. Actuators, B 2016, 234, 273–277. 10.1016/j.snb.2016.04.032.

[ref19] Li X.; Ying Y. L.; Fu X. X.; Wan Y. J.; Long Y. T. Single-Molecule Frequency Fingerprint for Ion Interaction Networks in a Confined Nanopore. Angew. Chem., Int. Ed. Engl. 2021, 60, 24582–24587. 10.1002/anie.202108226. 34390607

[ref20] Liu S.-C.; Li M.-X.; Li M.-Y.; Wang Y.-Q.; Ying Y.-L.; Wan Y.-J.; Long Y.-T. Measuring a frequency spectrum for single-molecule interactions with a confined nanopore. Faraday Discuss. 2018, 210, 87–99. 10.1039/c8fd00023a. 29985499

[ref21] Wen C.; Dematties D.; Zhang S.-L. A Guide to Signal Processing Algorithms for Nanopore Sensors. ACS Sens. 2021, 6, 3536–3555. 10.1021/acssensors.1c01618. 34601866 PMC8546757

[ref22] Ying Y.-L.; Long Y.-T. Nanopore-Based Single-Biomolecule Interfaces: From Information to Knowledge. J. Am. Chem. Soc. 2019, 141, 15720–15729. 10.1021/jacs.8b11970. 31509414

[ref23] Fragasso A.; Schmid S.; Dekker C. Comparing Current Noise in Biological and Solid-State Nanopores. ACS Nano 2020, 14, 1338–1349. 10.1021/acsnano.9b09353. 32049492 PMC7045697

[ref24] Uram J. D.; Ke K.; Mayer M. Noise and bandwidth of current recordings from submicrometer pores and nanopores. ACS Nano 2008, 2, 857–872. 10.1021/nn700322m. 19206482

[ref25] Balijepalli A.; Ettedgui J.; Cornio A. T.; Robertson J. W. F.; Cheung K. P.; Kasianowicz J. J.; Vaz C. Quantifying short-lived events in multistate ionic current measurements. ACS Nano 2014, 8, 1547–1553. 10.1021/nn405761y. 24397836 PMC3943493

[ref26] Dunbar W. B. Comment on Accurate Data Process for Nanopore Analysis. Anal. Chem. 2015, 87, 10650–10652. 10.1021/acs.analchem.5b02281. 26414231

[ref27] Gu Z.; Ying Y.-L.; Cao C.; He P.; Long Y.-T. Accurate data process for nanopore analysis. Anal. Chem. 2015, 87, 907–913. 10.1021/ac5028758. 25514172

[ref28] Gu Z.; Ying Y.-L.; Cao C.; He P.; Long Y.-T. Reply to Comment on Accurate Data Process for Nanopore Analysis. Anal. Chem. 2015, 87, 10653–10656. 10.1021/acs.analchem.5b03225. 25514172

[ref29] Pedone D.; Firnkes M.; Rant U. Data analysis of translocation events in nanopore experiments. Anal. Chem. 2009, 81, 9689–9694. 10.1021/ac901877z. 19877660

[ref30] Lucas F. L. R.; Piso T. R. C.; Heide N. J.; Galenkamp N. S.; Hermans J.; Wloka C.; Maglia G. Automated Electrical Quantification of Vitamin B1 in a Bodily Fluid using an Engineered Nanopore Sensor. Angew. Chem., Int. Ed. Engl. 2021, 60, 22849–22855. 10.1002/anie.202107807. 34390104 PMC8518494

[ref31] Houghtaling J.; Ying C.; Eggenberger O. M.; Fennouri A.; Nandivada S.; Acharjee M.; Li J.; Hall A. R.; Mayer M. Estimation of Shape, Volume, and Dipole Moment of Individual Proteins Freely Transiting a Synthetic Nanopore. ACS Nano 2019, 13, 5231–5242. 10.1021/acsnano.8b09555. 30995394

