# Supplementary material for: Unbiased Data Analysis for the Parameterization of Fast Translocation Events through Nanopores
Source: ACS Omega. 2022 Jul 19;7(30):26040–6. doi: 10.1021/acsomega.2c00871 (PMC9352258; doi:10.1021/acsomega.2c00871)
Supplement: Supplementary file 1 — ao2c00871_si_001.pdf [file ao2c00871_si_001.pdf]

Supporting Information

Unbiased Data Analysis for the Parameterization of Fast Translocation Events through Nanopores

Florian L. R. Lucas[1,3]*, Kherim Willems[2], Matthijs J. Tadema[1], Katarzyna M. Tych[1], Giovanni Maglia[1], Carsten Wloka[1,4]*

[1]Groningen Biomolecular Sciences and Biotechnology Institute, University of Groningen, Groningen, 9712 CP Groningen, The Netherlands

[2]IMEC, Kapeldreef 75, B-3001 Leuven, Belgium

[3]Lab for Nanobiology, Department of Chemistry, KU Leuven, 3001 Leuven, Belgium

[4]Experimental Ophthalmology, Department of Ophthalmology, Charité – Universitätsmedizin Berlin, a corporate member of Freie Universität, Humboldt-University, the Berlin Institute of Health, Berlin, Deutschland

***Corresponding Authors:**

Florian L. R. Lucas – Email: flrlucas@gmail.com

Carsten Wloka – Email: c.wloka@rug.nl

Table of contents

Supporting Information 1. Equations deriving the residual current at any given time………………………………………………………..S3

Supporting Information 2. Equations deriving dwell time from the gNDF……………………………………………………………………S4

Supporting Information 3. Equations deriving event localisation from the gNDF…………………………………………………………S5

Supporting Information 4. Equations deriving the one-equidistant probability from the gNDF…………………………………………S6

Supporting Information 5. Equations deriving the minimal required sampling frequency from the gNDF…………………………..S7

Supplementary Figure 1. Nanopore traces at different filter frequencies ……………………………………………………………………S8

Supplementary Figure 2. Difference in dwell time estimation when using the Full-Width at Half Maximum (FWHM) and back-mapped estimation………S9

Supplementary Figure 3. Histograms of fitted parameters for different filter frequencies..……………………………………………S10

Supporting Information 1

Simplify:

$$I_{res}(t) = \frac{I_B(t)}{I_o}$$

Under the assumptions that:

$$I_o = \frac{V_{ref}}{R_{open}}$$

$$I_B = \frac{V_{ref}}{R_{open} + R_{block}}$$

$$I_B(t) = \frac{V_{ref} - \mathrm{V_{ref}} * \exp\left(-\frac{t}{\tau_{system}}\right)}{R_{open} + R_{block}}$$

$$I_{res} = \frac{I_B}{I_o} = \frac{R_{open}}{R_{open} + R_{block}}$$

It follows:

$$I_{res}(t) = \frac{V_{ref} - \mathrm{V_{ref}} * \exp\left(-\frac{t}{\tau_{system}}\right)}{R_{open} + R_{block}} * \frac{R_{open}}{V_{ref}}$$

$$I_{res}(t) = \frac{R_{open}\left(V_{ref} - \mathrm{V_{ref}} * \exp\left(-\frac{t}{\tau_{system}}\right)\right)}{V_{ref}(R_{open} + R_{block})}$$

$$I_{res}(t) = \frac{R_{\mathrm{open}}}{R_{\mathrm{open}} + R_{block}} - \frac{R_{\mathrm{open}} * \exp\left(-\frac{t}{\tau_{\mathrm{system}}}\right)}{R_{\mathrm{open}} + R_{block}}$$

$$I_{res}(t) = I_{res} - I_{res} * \exp\left(-\frac{t}{\tau_{\mathrm{system}}}\right) = I_{res}\left(1 - \exp\left(-\frac{t}{\tau_{system}}\right)\right)$$

With parameters the open pore current (I_O), reference voltage (V_{ref}), resistance of the open pore (R_{open}), blocked pore current (I_B), resistance of the blocked pore (R_{block}), RC-time constant of the system (τ_{system}) and the residual current (I_{res}).

Supporting Information 2

Find Δt where the following supposition hold:

$$\Delta t \cdot h = \int_{-\infty}^{+\infty} f(t)dt$$

Using the following definitions:

$$\Delta t = t_1 - t_0$$

$$\int_{-\infty}^{+\infty} f(t)dt = 1$$

Under the assumption that:

$$h = \left[2\sigma\Gamma\left(\frac{1}{\beta} + 1\right)\right]^{-1}, \qquad \text{height } (h) \text{ is equal to the maximum} - \text{ordinate}$$

It follows:

$$\Delta t = (t_1 - t_0) = 2\sigma\Gamma\left(\frac{1}{\beta} + 1\right)$$

where t_0 and t_1 are the event start (0) and end (1) times respectively, h is the event height and $f(t)$ is the generalised Normal Distribution Function at any given time t, β is the shape parameter and σ is its standard deviation.

Therefore, the limit where the shape (β) parameter reaches infinity is given as follow:

$$\lim_{\beta \to \infty} 2\sigma\Gamma\left(\frac{1}{\beta} + 1\right) = 2\sigma$$

Supporting Information 3

<u>Find $t_1 \geqq \mu$ such that:</u>

$$\mathrm{t}_0 + \Delta t \geq \mu$$

<u>Under the assumption that:</u>

$$t_0 = Q(p)$$

<u>It follows:</u>

$$Q(p) + \Delta t \geq \mu$$

$$Q(p) + \Delta t - \mu \geq 0$$

where Δt is the total event time, t_0 and t_1 are the event start (0) and end (1) times respectively, $Q(p)$ is the quantile function of the generalised Normal Distribution Function (gNDF) at probability p, μ is the localisation of the gNDF.

Supporting Information 4

Simplify:

$$\nu(\mu - W_h) \geq 1 - \nu(t_1 + W_h)$$

Using the following definitions:

$\nu(x;\sigma,\beta,\mu) = \exp\left(-\left(\frac{|x-\mu|}{\sigma}\right)^{\beta}\right),$ *the probability function of the gNDF*

$W_h = \sigma\sqrt[\beta]{\ln 2},$ *the half width at half maximum*

$$\nu(\mu - W_h) = \frac{1}{2}$$

Under the assumptions that:

$t_1 = \Delta t + Q(p),$ $with\ Q(p) = t_0$

It follows:

$$\nu(\mu - W_h) \geq 1 - \nu(t_1 + W_h) \Longrightarrow$$

$$\nu(t_1 + W_h) \geq \frac{1}{2} \Longrightarrow$$

$$\nu(Q(p) + \Delta t + W_h) \geq \frac{1}{2} \Longrightarrow$$

$$\exp\left(-\left(\frac{|Q(p) + \Delta t + W_h - \mu|}{\sigma}\right)^{\beta}\right) \geq \frac{1}{2} \Longrightarrow$$

$$\ln 2 \geq \left(\frac{|Q(p) + \Delta t + W_h - \mu|}{\sigma}\right)^{\beta} \Longrightarrow$$

$$\sigma\sqrt[\beta]{\ln 2} \geq |Q(p) + \Delta t + W_h - \mu|$$

We can remove the absolute, as per Supplementary Information 3, resulting:

$$0 \geq Q(p) + \Delta t - \mu$$

where Δt is the total event time, t_0 and t_1 are the event start (0) and end (1) times respectively, $Q(p)$ is the quantile function of the generalised Normal Distribution Function (gNDF) at probability p. μ is the localisation, β is the shape parameter, and σ is its standard deviation of the gNDF. W_h and $\nu(x)$ are the half width at half maximum and the probability function of the gNDF respectively.

Supporting Information 5

Find x *where* $g(\mu) - g(x) = p$

Using the following:

$$g(x;\sigma,\beta,\mu) = \left[2\sigma\Gamma\left(\frac{1}{\beta}+1\right)\right]^{-1} \exp\left(-\left(\frac{|x-\mu|}{\sigma}\right)^{\beta}\right)$$

It follows:

$$g(\mu) - p = g(x) \Longrightarrow$$

$$\left[2\sigma\Gamma\left(\frac{1}{\beta}+1\right)\right]^{-1} - p = \left[2\sigma\Gamma\left(\frac{1}{\beta}+1\right)\right]^{-1} \exp\left(-\left(\frac{|x-\mu|}{\sigma}\right)^{\beta}\right) \Longrightarrow$$

$$1 - p * 2\sigma\Gamma\left(\frac{1}{\beta}+1\right) = \exp\left(-\left(\frac{|x-\mu|}{\sigma}\right)^{\beta}\right) \Longrightarrow$$

$$-\ln\left(1 - p * 2\sigma\Gamma\left(\frac{1}{\beta}+1\right)\right) = \left(\frac{|x-\mu|}{\sigma}\right)^{\beta} \Longrightarrow$$

$$|x-\mu| = \sigma \sqrt[\beta]{-\ln\left(1 - p * 2\sigma\Gamma\left(\frac{1}{\beta}+1\right)\right)} \Longrightarrow$$

$$x = \mu \pm \sigma \sqrt[\beta]{-\ln\left(1 - p * 2\sigma\Gamma\left(\frac{1}{\beta}+1\right)\right)}$$

$g(x)$ is the probability density function of the generalised Normal Distribution Function (gNDF). μ is the localisation, β is the shape parameter, and σ is its standard deviation of the gNDF. W_h and $\nu(x)$ are the half width at half maximum and the probability function of the gNDF respectively. p is the probability threshold defining the start time of an event.

Supplementary Figures

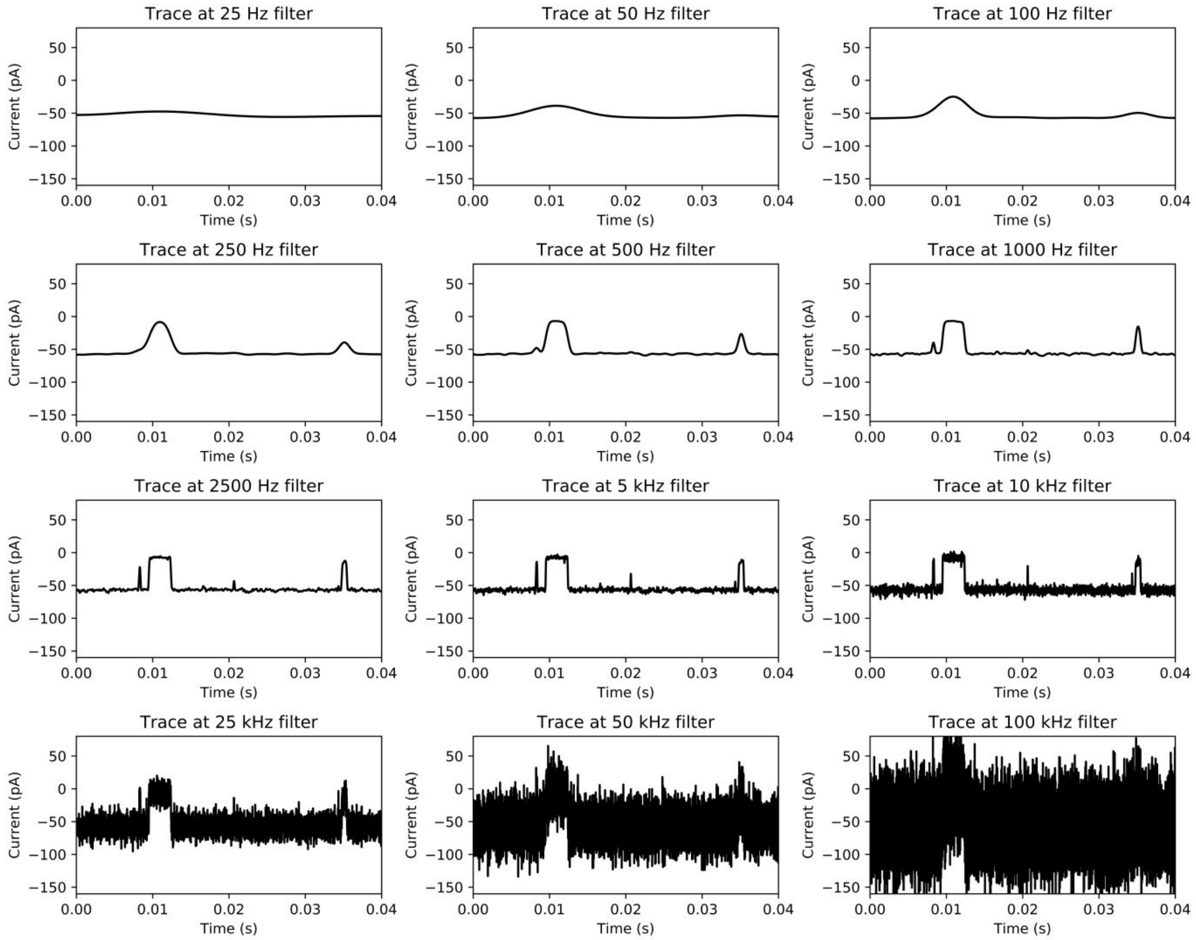

Supplementary Figure 1. Nanopore traces at different filter frequencies. Raw current trace of 10 µM (added to *cis*) of a penta-peptide (YAGFL) measured in 1 M KCl, pH 4.2 buffered using 15 mM citric acid and bis-tris-propane, under an applied potential of negative 100 mV (*cis*-*trans*) at a sampling frequency of 500 kHz with a recording bandwidth of 100 kHz using fragaceatoxin C modified with a tryptophan at position 13 (G13W-FraC). Experiments were performed using an Axon Axopatch 200B amplified (Molecular Devices) coupled to an Axon Digidata 1550B (Molecular Devices). Each panel represents the same trace, however, each has a digital Gaussian filter applied with a filter frequency of 25 Hz, 50 Hz, 100 Hz, 250 Hz, 500 Hz, 1000 Hz, 2500 Hz, 5 kHz, 10 kHz, 25 kHz, 50 kHz, or 100 kHz.

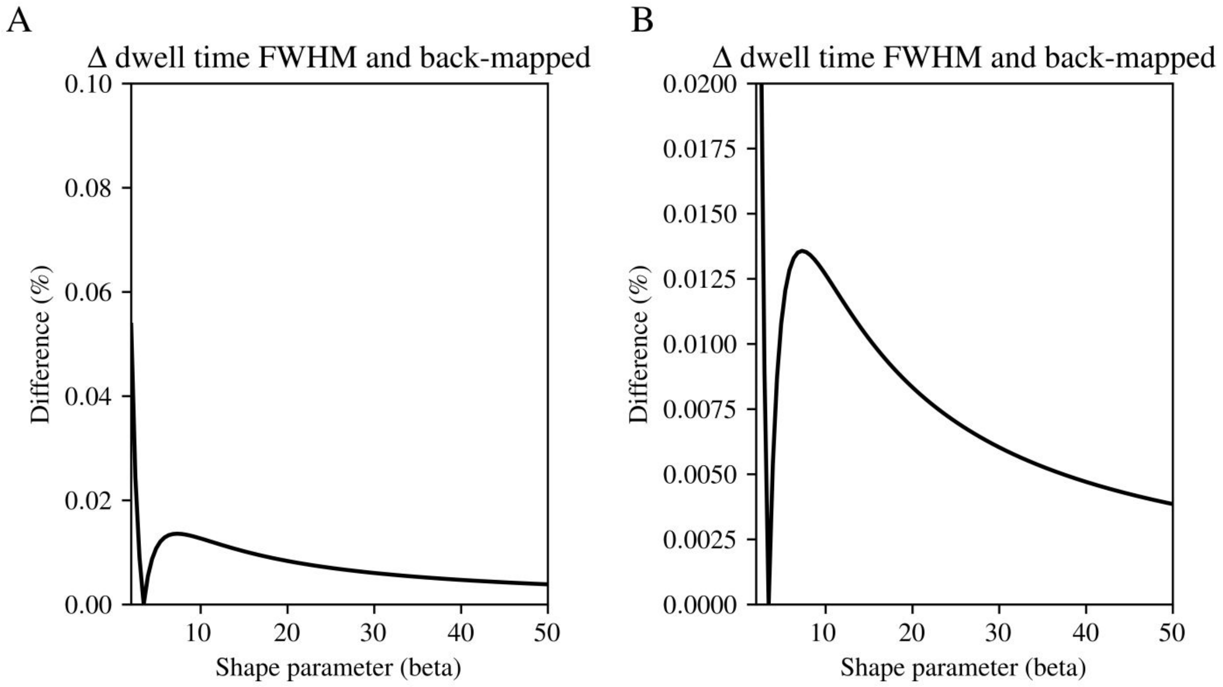

Supplementary Figure 2. Difference in dwell time estimation when using the Full-Width at Half Maximum (FWHM) and back-mapped estimation. Each figure shows the difference between the FWHM and back mapped dwell time estimation, equation 6 and 8 of the main manuscript respectively. **A**: difference between equation 6 and 8 of the main manuscript, with increasing shape parameter (beta). **B**: shows the same graph as A, however, the Y-axis is scaled between 0 and 0.02%**.**

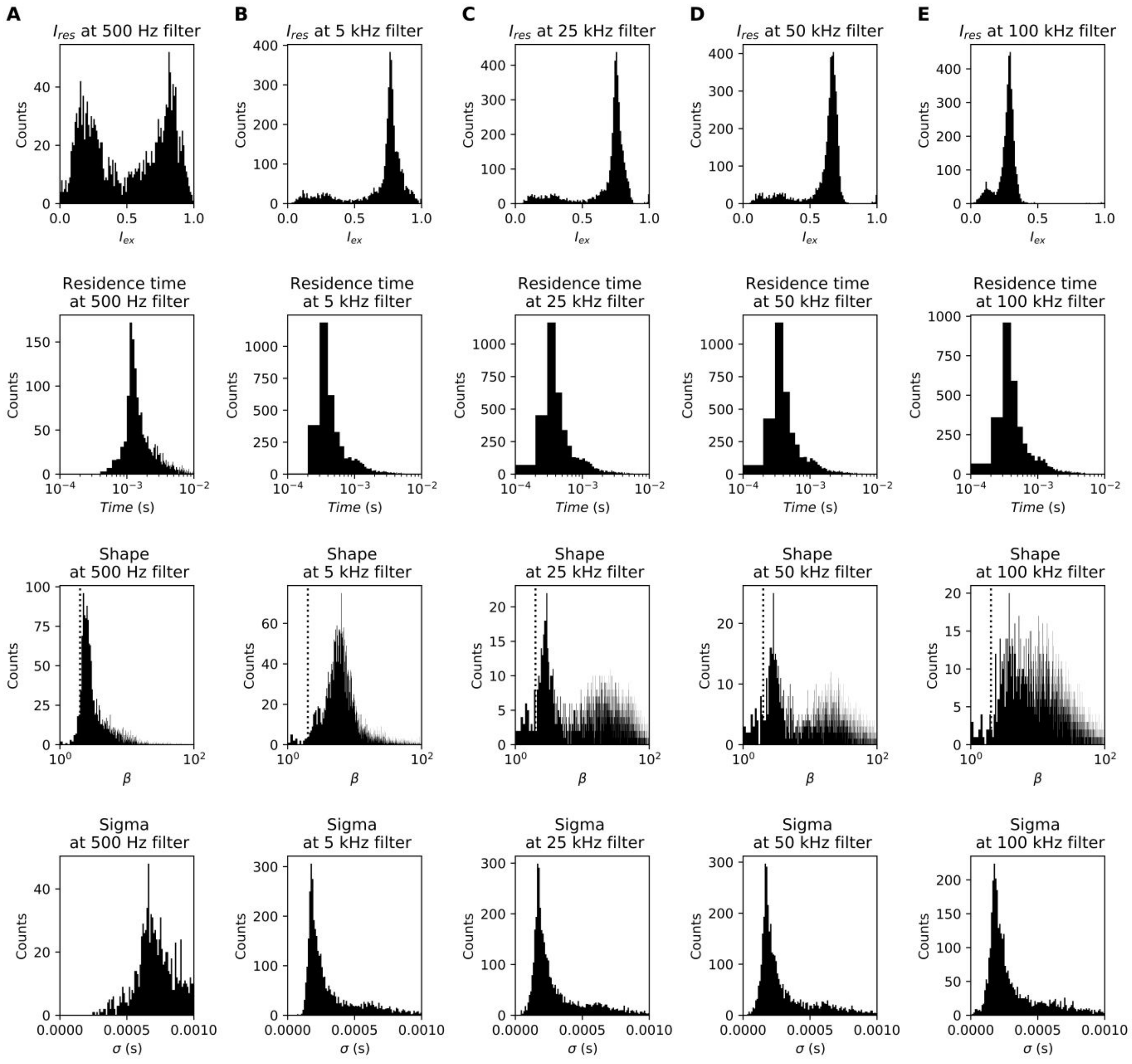

Supplementary Figure 3. Histograms of fitted parameters for different filter frequencies. All panels represent the same data where a 10 µM (added to *cis*) of penta-peptide (YAGFL) was measured in 1M KCl, pH 4.2 buffered using 15 mM citric acid and bis-tris-propane, under an applied potential of negative 100 mV (*cis-trans*) at a sampling frequency of 500 kHz with a recording bandwidth of 100 kHz using fragaceatoxin C modified with a tryptophan at position 13 (G13W-FraC). Experiments were performed using an Axon Axopatch 200B amplified (Molecular Devices) coupled to an Axon Digidata 1550B (Molecular Devices). All events were localised using a 5 kHz Gaussian filter, and subsequently characterised using a Gaussian filter at the filter frequency as described above each graph. A-E: Top graph: the excluded current ($\boldsymbol{I_{ex}}$) of each observed event ($\boldsymbol{F_{s}}_{,\text{event}}$ $\leq \mathbf{500}$ kHz) binned in 200 residual current bins evenly distributed between 0 and 1 $\boldsymbol{I_{ex}}$. The second row: a histogram of the residence time of events for different Gaussian filter frequencies (see title in each panel). The third row shows a histogram of the estimated shape parameter (β). The final row shows a histogram of the sigma (σ) parameter as fitted for all events.
